# Supplementary material for: Consequences of Thermal Variation during Development and Transport on Flight and Low-Temperature Performance in False Codling Moth (Thaumatotibia leucotreta): Fine-Tuning Protocols for Improved Field Performance in a Sterile Insect Programme
Source: Insects. 2022 Mar 23;13(4):315. doi: 10.3390/insects13040315 (PMC9030207; doi:10.3390/insects13040315)
Supplement: Supplementary file 1 [file insects-13-00315-s001.zip › insects-1626991-supplementary.pdf]

Supplementary materials

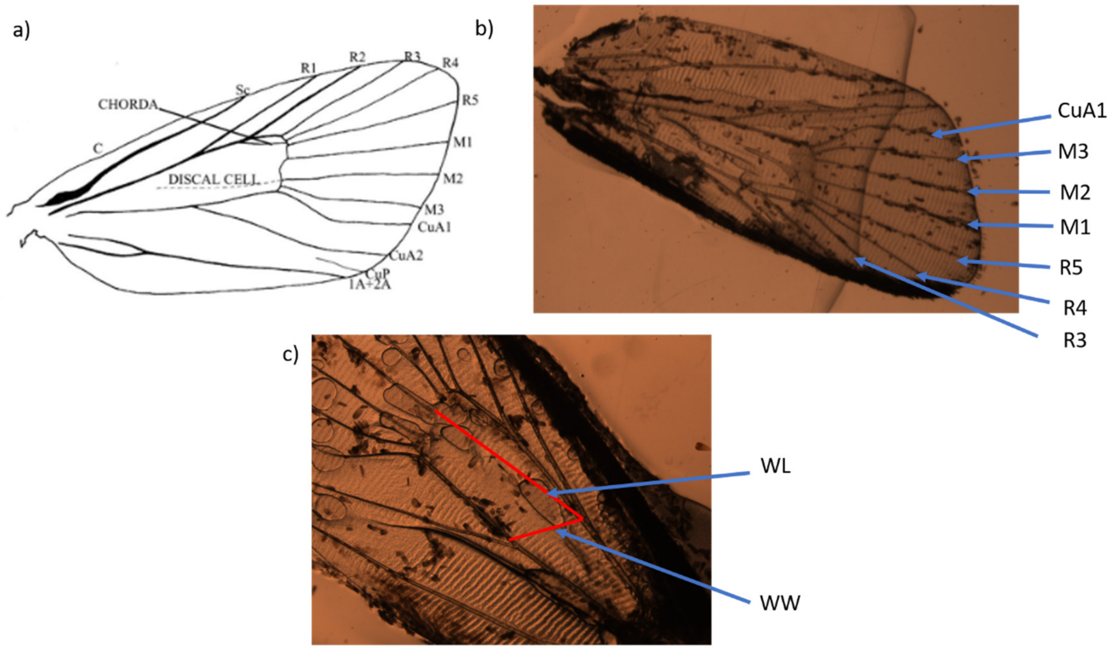

Figure S1: a) Wing venation of *Thaumatotibia leucotreta* adapted from [65] (D-discal cell; M-Media, R-Radius, Sc-Subcosta; CuA1-1st anterior cubitus; CuA2-2nd anterior cubitus; CuP-posterior cubitus) b) overall and c) zoomed picture of a *T. leucocreta* wing indicating the areas used as a measure of wing width (WW) and wing length (WL).

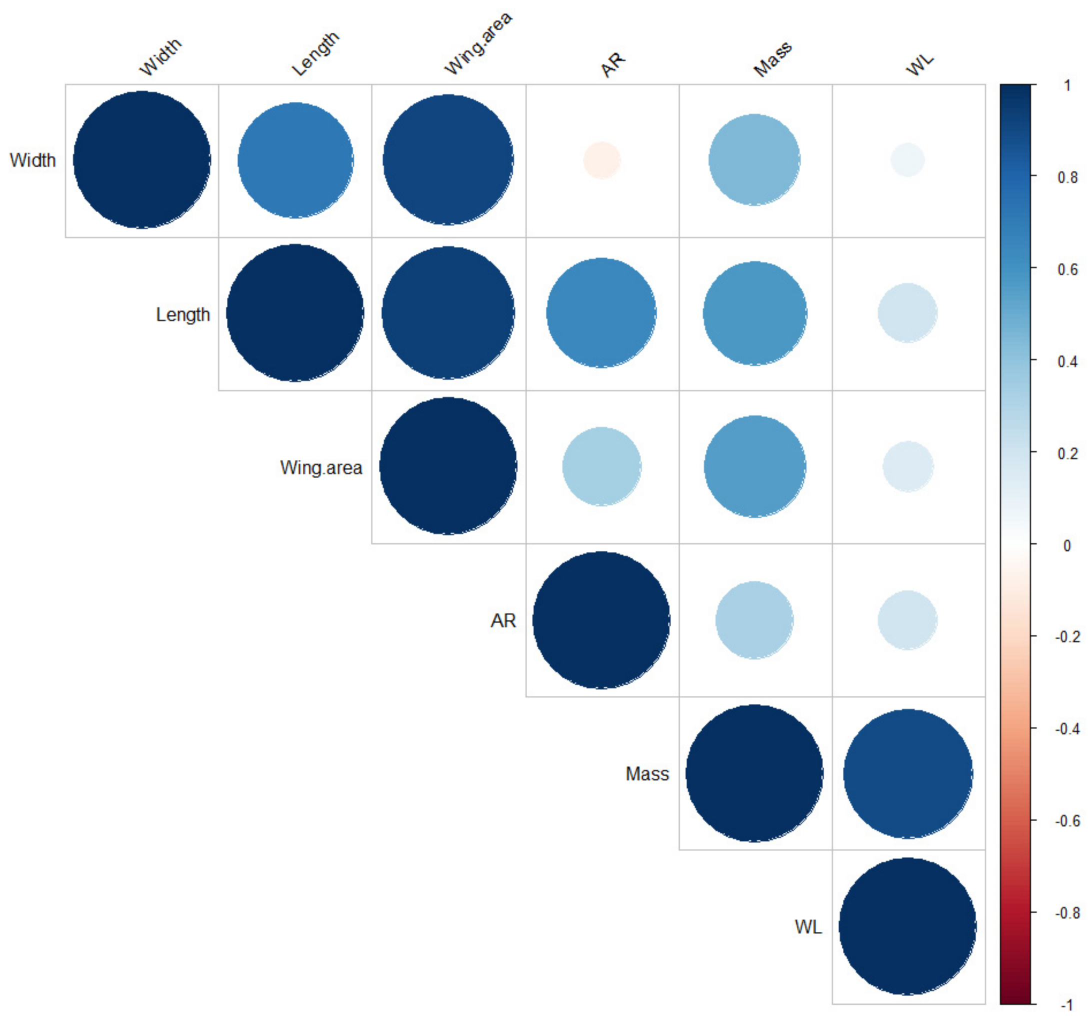

Figure S2: Correlation matrix comparing the morphological parameters (mass, wing width (Width), wing length (Length), wing area (Wing.area), wing loading (WL) and aspect ratio (AR)) measured from false codling moth (FCM) that underwent flight performance tests after developmental thermal acclimation. Positive numbers represent positive correlations and negative numbers represent negative correlations. Larger and darker circles represent significant correlations.
